# Influence of Leavening Agent on the Stability of Bioactive Compounds and Antioxidant Capacity of Gluten-Free Bread with Beetroot By-Product

**DOI:** 10.3390/molecules31040741

**Published:** 2026-02-21

**Authors:** Carmen Molina-Montero, Marta Igual, Javier Martínez-Monzó, Purificación García-Segovia

**Affiliations:** Programa de Doctorado en Ciencia, Tecnología y Gestión Alimentaria, i-Food Group, IIA-FoodUPV, Universitat Politècnica de València, Camino de Vera s/n, 46022 Valencia, Spain; mamomon3@doctor.upv.es (C.M.-M.); xmartine@tal.upv.es (J.M.-M.); pugarse@tal.upv.es (P.G.-S.)

**Keywords:** crumb, crust, betacyanins, betaxanthins, 3D food printing

## Abstract

Beetroot by-product (BBP), an industrial residue rich in bioactive compounds, offers a sustainable solution to reduce food waste while enhancing the nutritional profile. The aim of the study was to evaluate the effect of different leavening agents (baking powder and baker’s yeast) and geometry (rectangular and oval) on bioactive compound stability and antioxidant capacity when incorporating beetroot by-products into gluten-free bread formulations. Rectangular and oval-shaped gluten-free breads were produced using 3D printing. Moisture content, pH, color parameters, bioactive compounds (betalains and phenolic compounds), and antioxidant activity were analyzed in both crust and crumb. BBP addition significantly increased total phenolic content, antioxidant capacity, and betalain content in all formulations. Breads with baker’s yeast exhibited higher bioactive retention due to acidic pH levels that favor phenolic and betanin stability. Bread with baking powder showed a higher retention of betaxanthins (yellow pigments), while those with baker’s yeast retained betacyanins (red-violet pigments). Oval geometry improved moisture retention and bioactive preservation due to reduced surface exposure. This research demonstrates the feasibility of developing nutritionally enhanced gluten-free products using additive manufacturing. Bread enriched with beetroot by-product and baker’s yeast represents a suitable option to improve functionality and pigment retention while valorizing industrial waste.

## 1. Introduction

Global population growth has intensified food production and processing, leading to an exponential increase in the generation of agro-industrial by-products [1]. Food waste represents one of the greatest challenges in the current food system, with approximately one-third of the food produced worldwide being lost or wasted each year [2]. This problem has not only economic implications, but also environmental and social ones, contributing to greenhouse gas emissions and the inefficient use of natural resources. This scenario has driven international initiatives, such as the United Nations Sustainable Development Goals, whose target 12.3 sets a goal of halving per capita food waste and reducing losses in production and supply chains by 2030 [3]. In this context, the valorization of food industry by-products emerges as a key strategy within the circular economy paradigm, allowing materials traditionally considered waste to be transformed into value-added ingredients.

The valorization of beetroot by-products is particularly relevant considering the volume of processing worldwide. The European Union leads global beetroot production, accounting for approximately 70% of the total. During industrial processing for juice extraction, it is estimated that between 35 and 40% of the original biomass is converted into by-products, mainly in the form of pulp and peel [4]. These by-products are frequently destined for animal feed or composting [5].

Beetroot (*Beta vulgaris* L.) is a dicotyledonous plant belonging to the *Chenopodiaceae* family and is one of the oldest vegetables in the world [6]. It stands out among vegetables for its phytochemical profile, being particularly renowned for its content of betalains, a group of water-soluble nitrogenous pigments. These molecules are subdivided into two main categories: betacyanins, responsible for red-violet hues and whose main representative is betanin, and betaxanthins, which provide yellow-orange hues, with vulgaxanthin being their main component [4,7]. Beetroot has beneficial health properties, including antioxidant, anti-inflammatory, anti-cancer, and anti-diabetic activity, as well as hepatoprotective, hypotensive, and healing effects [8,9]. In addition, beetroot contains significant amounts of phenolic compounds, including phenolic acids and flavonoids, which contribute synergistically to its total antioxidant capacity [4,10].

BBP, a residue resulting from the juice extraction process, is rich in fiber and contains substantial amounts of phenolic compounds and betalains. This by-product is not fully utilized and could be considered an important source of natural antioxidants that improve food quality [11]. However, incorporating these by-products into food formulations presents challenges related to the stability of bioactive compounds. Both betalains and phenolic compounds are highly unstable under different conditions, such as pH, light, temperature, and the presence of oxygen. Some of these factors can cause significant changes in the chemical structure of bioactive compounds, leading to the loss of their activity and potential health benefits [12].

Several studies have explored the incorporation of BBP into different food matrices, such as snacks [5,13], cookies [14,15,16], yogurt [17], mayonnaise [11], or meringue [1], demonstrating not only technological feasibility but also improvements in the nutritional and functional profile of the resulting products.

This trend toward incorporating bioactive ingredients is part of the growing interest in functional foods, defined as those that, in addition to their basic nutritional value, provide additional health benefits or reduce the risk of chronic diseases [18]. Demand for these products has increased significantly in recent decades, driven by consumers who are increasingly aware of the relationship between diet and health, as well as a preference for natural ingredients over synthetic additives [5,6].

Gluten-free bakery products represent a promising target for functional enrichment. These products, intended for people with celiac disease or non-celiac gluten sensitivity, traditionally have nutritional limitations compared to their conventional counterparts, including lower fiber, protein, and micronutrient content [19]. Fortification with bioactive-rich by-products, such as BBP, offers the potential to improve the deficient nutritional profile of these products. However, this incorporation poses particular technological challenges related to the absence of the gluten network, which makes it difficult to obtain adequate structures, especially when using emerging technologies such as 3D food printing.

3D food printing is an emerging technology based on the layer-by-layer deposition of food material through extrusion. Unlike conventional molding, this technology enables precise control over geometry, layer thickness, and internal structure, allowing the modulation of heat and mass transfer phenomena during baking. The surface-to-volume ratio and internal filling design directly affect moisture migration, which in turn influences the degradation of heat-sensitive compounds such as betalains [20]. This level of structural control is difficult to achieve with conventional processes and offers new possibilities for optimizing the retention of bioactive compounds in baked goods.

The objective of this study was to evaluate the effect of incorporating beetroot by-product into gluten-free breads made using 3D printing on physicochemical parameters and the content of bioactive compounds. Specifically, the influence of the type of leavening agent (baking powder vs. baker’s yeast), the geometry of the product (rectangular vs. oval), and the addition of BBP on the moisture content, pH, color parameters, total phenolic content, antioxidant activity, and betalain profile (betanin and vulgaxanthin) of the final product after baking was analyzed. Since the type of leavening agent determines the pH of the dough, and pH is a key factor in betalain stability, differences in bioactive compound retention between leavening systems were expected. Additionally, the geometry of 3D-printed samples may influence heat exposure and moisture distribution during baking, potentially affecting the preservation of bioactive compounds in crust and crumb.

## 2. Results and Discussion

### 2.1. Physicochemical and Bioactive Characterization of Gluten-Free Dough

Table 1 shows the physicochemical properties and color parameters of gluten-free dough formulated with different leavening agents and BBP addition. The multifactorial ANOVA revealed a significant interaction between the leavening agent and BBP addition for pH values (*p* < 0.05). Doughs prepared with baking powder exhibited neutral pH values regardless of by-product incorporation. In contrast, doughs leavened with baker’s yeast showed more acidic pH values, with the addition of BBP resulting in a slight increase. The pH differences between leavening agents are attributed to the fact that baking powder is a mixture of alkaline and acidic compounds that generates a neutral pH, whereas baker’s yeast produces organic acids during fermentation, resulting in dough acidification [21].

Moisture content was significantly affected by the leavening agent (*p* < 0.05). Formulations with baker’s yeast retained slightly higher moisture compared to those with baking powder. This difference may be attributed to the fermentation process, during which yeast metabolic activity can modify the dough matrix structure and water-holding capacity through the production of metabolites such as glycerol, which acts by preventing dehydration and thereby enhancing water retention in the dough matrix [22]. BBP addition did not significantly (*p* > 0.05) influence moisture content.

Regarding color coordinates, the multifactorial ANOVA revealed a significant interaction between the leavening agent and the addition of BBP for the L* and a* values (*p* < 0.05). Formulations without BBP showed high luminosity values, indicating bright doughs, with baking powder formulations being significantly (*p* < 0.05) lighter than those with baker’s yeast. In contrast, BBP incorporation reduced luminosity, resulting in darker doughs. In relation to the a* parameter, formulations without BBP exhibited negative values, indicating greenish hues, with this effect being significantly (*p* < 0.05) more pronounced with baking powder than with baker’s yeast. BBP addition increased a* values toward the positive range, reflecting an intense reddish coloration, and was significantly (*p* < 0.05) higher with baker’s yeast. Both the reduction in L* and the increase in a* are attributed to the betalains present in BBP, which confer a characteristic red-purple coloration to the doughs. For the b* parameter, both leavening agent and BBP addition showed significant main effects (*p* < 0.05), with BBP being the most influential factor. Formulations with baker’s yeast exhibited higher b* values compared to those with baking powder, indicating a slightly more yellowish hue. BBP incorporation reduced b* values from 8.6 to 5.2, suggesting that the intense red-purple pigmentation from betalains partially masks the yellowish tones typically associated with gluten-free flour formulations.

**Figure 1:**
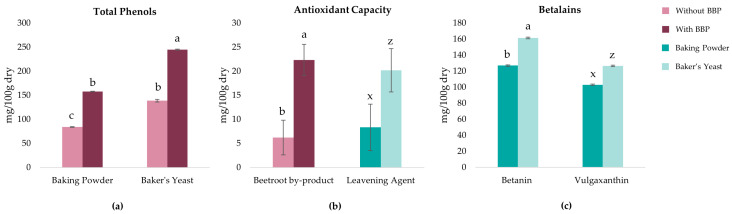
Effect of leavening agent (baking powder vs. baker’s yeast) and beetroot by-product (BBP) on bioactive compounds: (**a**) total phenolic content showing interaction effect between leavening agent and BBP addition; (**b**) antioxidant capacity showing main effects of BBP and leavening agent; (**c**) betalain content (betanin and vulgaxanthin) in doughs with BBP. Error bars represent standard error. Different letters indicate significant differences (*p* < 0.05) according to Fisher’s LSD test.

The incorporation of BBP into gluten-free dough formulations was evaluated for its impact on total phenolic content (TPC), antioxidant capacity (AC), and betalain profile. The multifactorial ANOVA revealed a significant interaction between leavening agent and BBP addition for TPC (*p* < 0.05) (Figure 1a). Formulations without BBP showed different TPC depending on the leavening agent used, with baker’s yeast formulations containing significantly (*p* < 0.05) higher TPC than baking powder formulations. BBP incorporation increased TPC in both leavening systems; however, this increase was greater in baker’s yeast formulations, representing increases of approximately 88%. The higher TPC observed in baker’s yeast formulations, even without BBP addition, may be attributed to the enzymatic activity during fermentation, which can release bound phenolic compounds from the flour matrix through hydrolytic reactions [23,24,25]. The enhanced phenolic retention when combining BBP with baker’s yeast may be attributed to the acidic pH generated during fermentation (pH ~5.8). According to Pasquet et al. [26], acidic conditions are particularly favorable for maintaining the stability of phenolic compounds, which remain stable in acidic pH but undergo degradation in alkaline conditions through auto-oxidation mechanisms.

For AC (Figure 1b), both BBP addition and leavening agent showed significant main effects (*p* < 0.05), with no significant interaction between factors. The BBP addition increased AC. Similarly, formulations with baker’s yeast exhibited significantly higher AC compared to those with baking powder, following the same trend observed for TPC. A strong positive correlation was found between TPC and AC (r = 0.970, *p* < 0.05).

Regarding the betalains content (betanin and vulgaxanthin) (Figure 1c), it was found that baker’s yeast significantly (*p* < 0.05) favors greater pigment retention compared to baking powder. These results suggest that the conditions generated during fermentation with baker’s yeast are less aggressive for betalain stability than those generated by baking powder. Furthermore, regardless of the leavening agent used, betanin remained the major component, showing concentrations 23% and 28% higher than those of vulgaxanthin for doughs with baking powder and baker’s yeast, respectively. Correlation analysis revealed strong associations between betalain content and all color parameters. Betalains showed strong positive correlations with a* values (betanin: r = 0.992; vulgaxanthin: r = 0.995, *p* < 0.05), confirming their contribution to the red coloration. Strong negative correlations were observed with L* values (betanin: r = −0.978; vulgaxanthin: r = −0.983, *p* < 0.05) and b* values (betanin: r = −0.930; vulgaxanthin: r = −0.939, *p* < 0.05), indicating that betalain incorporation reduces both luminosity and yellow tones, resulting in the characteristic red-purple coloration of BBP containing doughs. Additionally, betalain content was strongly associated with antioxidant capacity (betanin: r = 0.996; vulgaxanthin: r = 0.997, *p* < 0.05) and total phenolic content (betanin: r = 0.999; vulgaxanthin: r =0.998, *p* < 0.05), demonstrating that betalains contribute significantly to both the visual characteristics and bioactive profile of the doughs.

### 2.2. pH and Moisture Content of Crust and Crumb

**Figure 2:**
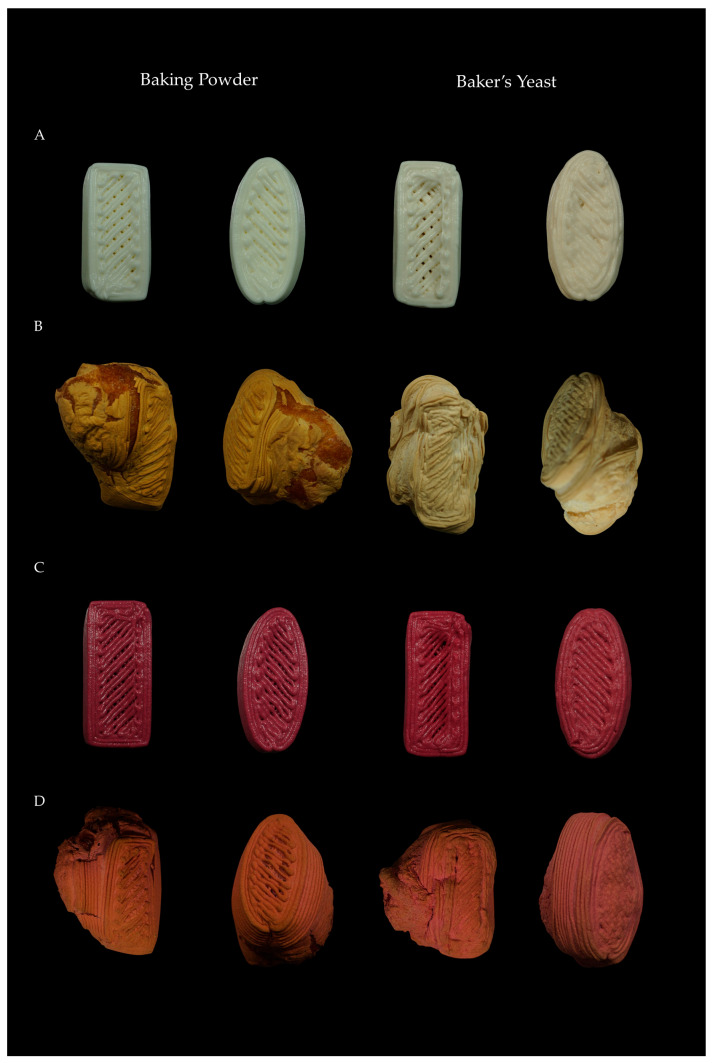
3D-printed gluten-free breads before and after baking. (**A**) Control formulation before baking, (**B**) control formulation after baking, (**C**) formulation with beetroot by-product before baking, (**D**) formulation with beetroot by-product after baking. For each formulation, samples are shown from left to right: baking powder with rectangular geometry, baking powder with oval geometry, baker’s yeast with rectangular geometry, and baker’s yeast with oval geometry.

Figure 2 shows the visual appearance of 3D-printed gluten-free breads before and after baking, for the control formulation and the formulation with added BBP made with different leavening agents and geometries.

The crust and crumb, although derived from the same original dough, develop different physicochemical properties during baking due to differences in heat exposure, moisture loss, and volatilization of compounds [27].

**Figure 3:**
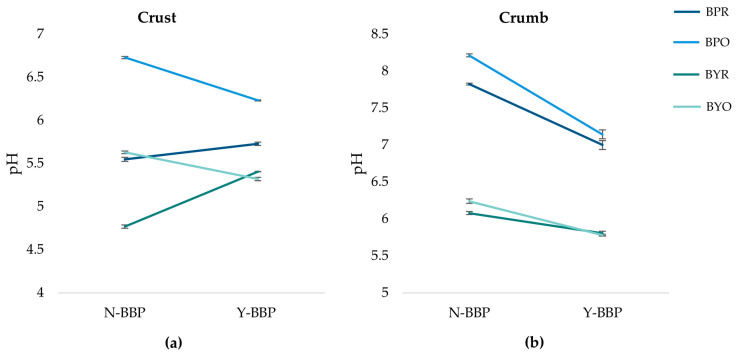
Effect of leavening agent (BP: baking powder; BY: baker’s yeast), geometry (R: rectangular; O: oval), and beetroot by-product (BBP) (N-BBP: without beet by-product; Y-BBP: with beet by-product) addition on pH in crust (**a**) and crumb (**b**) of 3D-printed gluten-free bread. The three-way interaction was significant for both crust and crumb (*p* < 0.05). Error bars represent standard error.

Figure 3 shows the significant triple interaction (*p* < 0.05) between leavening agent, geometry, and BBP (*p* < 0.05) for the pH of both the crust and the crumb. In the crust (Figure 3a), samples with baking powder had a higher pH than samples with baker’s yeast in both geometries, maintaining the differences previously observed in the dough. However, these pH differences were dependent on geometry, being more pronounced in oval shapes than in rectangular ones. The oval shapes without BBP addition showed higher pH values than the samples with rectangular geometry, regardless of the leavening agent used. On the other hand, the addition of BBP had opposite effects depending on the geometry. In oval shapes, it tended to decrease, while in rectangular shapes it increased.

In the crumb (Figure 3b), a clear difference was observed between the two leavening systems, with the samples containing baking powder having a higher pH compared to the samples containing baker’s yeast, which had a lower pH. The crumb had higher pH values than the crust in all formulations, indicating that the baking process contributes to reducing the pH in the crust. It was observed that the samples without BBP with oval geometry for both leavening agents had higher pH values than the rectangular samples, following the same trend as in the crust. When BBP was added, both the samples with baking powder and those with baker’s yeast decreased in pH, with a more pronounced effect in the samples with baking powder. This may be due to the organic acid content of BBP, which is retained in the crumb due to the moderate temperature conditions [28].

These results are consistent with previous studies showing that the type of leavening agent significantly affects the pH of the final product. Zolfaghari et al. [29] observed that lavash bread made with sodium bicarbonate had a higher pH than that made with baker’s yeast or sourdough, where prolonged fermentation progressively reduced the pH.

**Figure 4:**
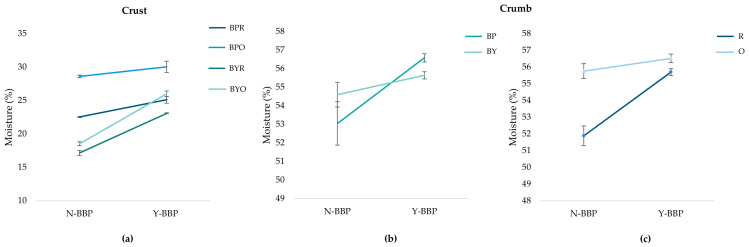
Effect of leavening agent (BP: baking powder; BY: baker’s yeast), geometry (R: rectangular; O: oval), and beetroot by-product (BBP) (N-BBP: without beet by-product; Y-BBP: with beet by-product) addition on moisture content in crust (**a**) and crumb (**b**,**c**) of 3D-printed gluten-free bread. Graphs show significant interactions: (**a**) leavening agent × geometry × BBP in crust; (**b**) leavening agent × BBP in crumb; (**c**) geometry × BBP in crumb (*p* < 0.05). Error bars represent standard error.

During the baking of gluten-free breads, water evaporates rapidly from the surface layers, forming the crust, resulting in a significantly lower moisture content in the crust compared to the core of the crumb [27]. Figure 4 shows the significant triple interaction (*p* < 0.05) between the leavening agent, geometry, and BBP of the crust, and the significant double interactions (*p* < 0.05) between the leavening agent and BBP, and between geometry and BBP, of the crumb. In the crust (Figure 4a), samples with baking powder had a significantly higher moisture content (*p* < 0.05) than samples with baker’s yeast. This behavior is consistent with García-Hernandez et al. [30], who reported that baking powder retains more moisture than baker’s yeast due to differences in the structure developed during leavening. The addition of BBP increased the moisture content in all formulations, with a more pronounced increase in formulations with baker’s yeast and oval geometry. This effect is attributed to the hygroscopic properties of the components of BBP, particularly its dietary fiber content [4]. In formulations with baker’s yeast, the fermentation process may contribute to modifying the characteristics of the fiber, enhancing its interaction with water and improving moisture retention efficiency [31,32].

In the crumb, concerning the effect of the leavening agent and BBP (Figure 4b), the samples without BBP made with baker’s yeast had a higher moisture content than those made with baking powder. The addition of BBP increased the moisture content in both leavening systems, resulting in slightly higher values for the samples with baking powder. Regarding the effect of geometry and BBP (Figure 4c), oval shapes showed higher moisture content than rectangular shapes, both in the presence and absence of BBP. The oval shape, with less surface area exposed per unit volume, favors lower moisture loss during baking. The addition of BBP increased the moisture content in both geometries. These results indicate that the incorporation of BBP improves moisture retention in the crumb, regardless of the leavening agent or geometry used, which can contribute positively to maintaining the freshness of the product during storage [33].

### 2.3. Color Parameters in Crust and Crumb

**Figure 5:**
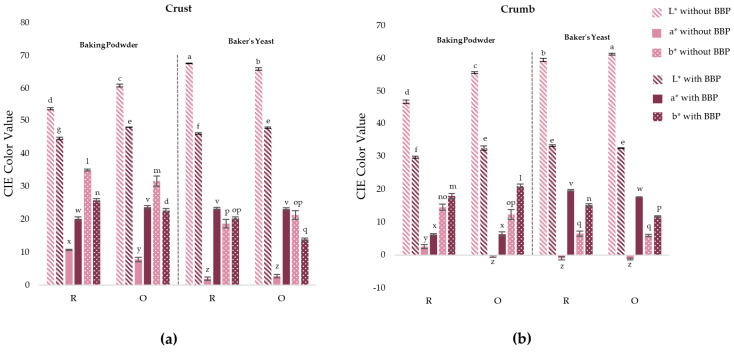
Effect of leavening agent (BP: baking powder; BY: baker’s yeast), geometry (R: rectangular; O: oval), and beetroot by-product (BBP) addition (with BBP; without BBP) on CIELab color parameters (L*, a*, and b*) in crust (**a**) and crumb (**b**) of 3D-printed gluten-free bread. The three-way interaction (leavening agent × geometry × BBP) was significant for all color parameters in both crust and crumb (*p* < 0.05). Different letters indicate significant differences among formulations within each parameter (*p* < 0.05). Error bars represent standard error.

Color is a fundamental quality attribute in baked goods, resulting from Maillard reactions and caramelization in the crust, as well as the presence of natural pigments in the ingredients [34]. Figure 5 shows the significant triple interactions (*p* < 0.05) between the leavening agent, geometry, and BBP for the color parameters L*, a*, and b* in the crust and crumb, and Table 2 shows photographs of the crumb for the different formulations.

In the crust (Figure 5a), samples without BBP and with baker’s yeast showed higher lightness and lower a* and b* values compared to samples with baking powder, as shown in Figure 2. This may be due to pH differences between the formulations. Maillard reactions are responsible for color development in the crust of baked products and are favored under alkaline pH conditions. The more acidic pH of samples with baker’s yeast limits the Maillard reaction, resulting in the lower formation of melanoidins and brownish-reddish and yellowish compounds, which leads to a lighter crust [35,36,37,38].

As can be seen in Figure 2, the addition of BBP produced a significant darkening (*p* < 0.05) of the crust, as well as a significant increase in the a* parameter, in all formulations. This is due to the betalains present in BBP, which impart reddish hues [4,7]. Regarding the b* parameter, in samples with baking powder it decreased, while in rectangular samples with baker’s yeast no significant changes were observed, although in oval ones a significant decrease (*p* < 0.05) was observed. This reduction may be due to the masking of yellow tones (both from Maillard and caramelization) by the red-violet pigments of betalains, resulting in a crust with a more reddish and less yellowish tonality.

The total color difference (ΔE) between leavening systems in the crust was significant (*p* < 0.05). In samples without BBP, the ΔE values between baking powder and baker’s yeast were very high (12.5 (oval) and 23.2 (rectangular)), indicating clearly perceptible color differences associated with different development of Maillard reactions as a function of pH. The addition of BBP reduced these differences (6.7 (rectangular) and 8.9 (oval)), suggesting that the pigmenting effect of betalains tends to homogenize the crust color, although differences perceptible by the human eye (>3) persist [39]. No significant differences (*p* > 0.05) were found between geometries (*p* > 0.05).

In the crumb (Figure 5b), samples without BBP showed the same behavior as in the crust, except for the b* parameter. Samples with baker’s yeast were significantly (*p* < 0.05) lighter than samples with baking powder, as observed in Table 2. This pattern, as in the crust, is associated with lower development of Maillard reactions at acidic pH in formulations with baker’s yeast. The a* parameter showed negative values (greenish hue) in most formulations, except in rectangular ones with baking powder, which showed low positive values. Regarding the b* parameter, samples with baking powder showed higher values than those with baker’s yeast, reflecting the greater development of yellow hues favored by alkaline pH, as can be observed in Table 2 [38].

The addition of BBP produced a darkening of the crumb in all formulations due to the incorporation of betalains. No significant differences (*p* > 0.05) were found between leavening agents, except in rectangular samples with baking powder, which were slightly darker, although the difference was minimal. This indicates that the pigmenting effect of BBP in the crumb is independent of the leavening system used. Additionally, it significantly increased a*, with a more pronounced increase in samples with baker’s yeast, obtaining a crumb with a reddish coloration (Table 2). In contrast, the b* parameter was significantly (*p* < 0.05) higher in samples with baking powder, obtaining a more yellowish coloration.

The ΔE values in the crumb were significant for both leavening agents and geometries (*p* < 0.05). In samples without BBP, the differences between baking powder and baker’s yeast were highly perceptible (8.8 (oval)–15.6 (rectangular)), being more pronounced in rectangular shapes. The addition of BBP maintained these differences at similar or higher values (14.3 (rectangular)–14.7 (oval)), in contrast to what was observed in the crust, where BBP reduced the chromatic differences between leavening agents. Regarding the effect of geometry, color differences were more pronounced in samples with baking powder without BBP (10.5), while in the other formulations, the differences were smaller, but still perceptible (2.6–4.7).

### 2.4. Bioactive Compounds in Crust and Crumb

The incorporation of BBP in baked products can improve their nutritional profile by providing bioactive compounds with nutritional properties. However, factors such as temperature, pH, water activity, light intensity, or chelating agents can affect the stability of betalains [40]. Table 3 presents the TPC and AC of the crust and crumb of 3D-printed gluten-free bread.

In the crust, the multifactorial ANOVA revealed significant double interactions (*p* < 0.05) between leavening agent and geometry, between leavening agent and BBP, and between geometry and BBP for TPC, and a significant triple interaction (*p* < 0.05) for AC. It was observed that samples with baker’s yeast presented higher TPC and AC than those with baking powder. This behavior can be attributed to the formation of phenolic compounds during the fermentation process and, therefore, to a greater antioxidant capacity, as detailed in Section 2.1. The rectangular sample showed the highest TPC, which can be attributed to its higher surface, resulting in greater crust area exposed to heat. The high temperatures in the crust favor the formation of new phenolic compounds through Maillard reactions and caramelization [41]. Furthermore, Maillard reaction products, particularly melanoidins, generated during baking may also contribute to the antioxidant capacity measured in the crust [42,43]. The addition of BBP significantly increased TPC both in formulations with baker’s yeast and in rectangular samples. AC also increased in all formulations, with this increase being more pronounced in samples with baker’s yeast. BBP is a rich source of phenolic compounds, mainly phenolic acids (ferulic acid, caffeic acid) and flavonoids, as well as betalains with antioxidant properties [4]. In the crumb, a significant triple interaction (*p* < 0.05) was observed among leavening agent, geometry, and BBP for TPC, and significant double interactions (*p* < 0.05) between leavening agent and shape and between leavening agent and beetroot for AC. The TPC and AC were significantly higher than those in the crust across all formulations. During baking, the temperatures reached in the crumb typically do not exceed 100 °C, making degradation of compounds less likely and favoring the retention of these thermosensitive compounds [44,45,46]. Samples with baker’s yeast showed higher TPC and AC than those with baking powder, maintaining the pattern observed in the crust. The addition of BBP significantly increased (*p* < 0.05) both parameters in all formulations, with this increase being more pronounced in samples with baker’s yeast and oval samples. The milder temperature conditions in the crumb favor the preservation of BBP phenolic compounds, with oval geometries being more protective due to their more compact structure and lower surface, which results in reduced heat exposure and better retention of thermosensitive bioactive compounds. In the crumb, a significant positive correlation was observed between TPC and AC (r = 0.778; *p* < 0.05), confirming that phenolic compounds contribute to antioxidant capacity when not subjected to high thermal degradation.

**Figure 6:**
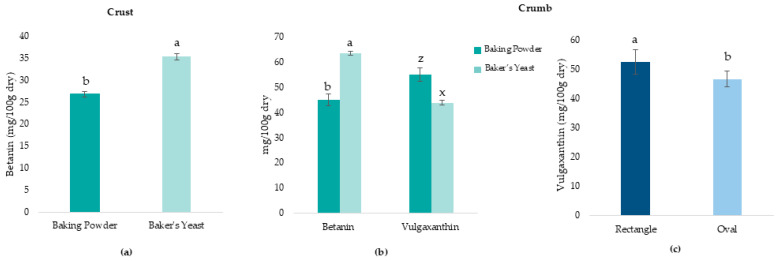
Effect of significant factors on betalain content in 3D-printed gluten-free bread with beetroot by-product addition. (**a**) Leavening agent effect on betanin content in crust; (**b**) Leavening agent effect on betanin and vulgaxanthin content in crumb; (**c**) Geometry effect on vulgaxanthin content in crumb. Different letters (a,b; z,x) indicate significant differences between groups (*p* < 0.05). Error bars represent standard deviation.

Figure 6 shows the significant factors and interactions (*p* < 0.05) of betanin and vulgaxanthin content in the crust and crumb.

In the crust (Figure 6a), the leavening agent factor had a significant effect (*p* < 0.05) on betanin content. Samples with baker’s yeast showed higher betanin content than those with baking powder. Regarding vulgaxanthin content, no significant effects (*p* > 0.05) of leavening agent, geometry, or their interaction were found, possibly due to greater thermal degradation of these pigments on the bread surface [47].

In the crumb, the effect of the leavening agent was significant for both betanin and vulgaxanthin content (Figure 6b). Betanin content was 1.7 and 1.8 times higher than in the crust for baking powder and baker’s yeast, respectively. Samples with baker’s yeast showed higher betalain content than baking powder. In contrast, vulgaxanthin content was significantly higher in samples with baking powder. Vulgaxanthin content was also significantly (*p* < 0.05) affected by the geometry factor (Figure 6c). Rectangular samples showed higher vulgaxanthin content. This can be related to their higher surface-to-volume ratio, which promotes greater moisture loss in the crumb (Figure 4c). Water activity significantly affects betalain stability, with lower water activity values being associated with reduced betalain degradation [48]. The lower moisture content in the crumb of rectangular pieces could therefore create a more favorable environment for vulgaxanthin preservation. This effect was significant only for vulgaxanthin, which is consistent with the reported lower stability of betaxanthins compared to betacyanins against environmental factors such as temperature and water activity [49].

It was observed that in both crust and crumb, total phenolic content and antioxidant activity were higher when BBP was added, especially in samples with baker’s yeast. This is due to the pH reduction in samples with baker’s yeast, which favors the retention of phenolic compounds [50]. The higher retention of betanins in the crumb of samples with baker’s yeast is also a pH effect. Samples with baker’s yeast, having a more acidic pH, preferentially retain betacyanins (red pigments), while samples with baking powder, which have a more alkaline pH, favor the retention of betaxanthins (yellow pigments) [51,52]. This pH–betanin relationship was confirmed by the strong negative correlation observed between these parameters (r = −0.970, *p* < 0.05), indicating that pH increase reduces the stability of betacyanins. The strong positive correlation between pH and vulgaxanthin (r = 0.821, *p* < 0.05) confirms that alkaline pH favors the retention of these pigments. These trends are further supported by the betalain retention values after baking relative to raw dough (Table 4). In all formulations, retention was higher in the crumb than in the crust, showing the greater thermal degradation at the bread surface. Betanin retention in the crust and crumb was similar across all samples, while vulgaxanthin retention was higher in baking powder samples than in baker’s yeast samples, confirming the differential pH-dependent stability between both betalain types.

The betalain results explain the chromatic differences observed in Table 2 and in the CIELab color parameters (Figure 5). Samples with baker’s yeast, which retained higher betacyanin content (red-violet pigments) due to acid pH, showed higher a* values and developed the characteristic reddish coloration in the crumb. Conversely, samples with baking powder, with higher betaxanthin content (yellow pigments) due to neutral pH, showed higher b* values and brownish-yellowish hues. This behavior demonstrates that the pH of the matrix not only affects color development through thermal reactions but also pigment stability.

## 3. Materials and Methods

### 3.1. Raw Materials

The bread dough was made with ingredients including a gluten-free flour mix of corn starch, rice flour, dextrose, vegetable fibers (psyllium and apple), a stabilizer (HPMC), egg powder, and salt, all sourced from Sinblat (Sinblat Alimentación Saludable S.L., Foios, Spain). Baker’s yeast (Saccharomyces cerevisiae), provided by Sosa Ingredients (Sosa Ingredients S.L.U., Barcelona, Spain), served as a leavening agent. Additionally, baking powder containing rice flour, sodium bicarbonate (E-550ii), and a pyrophosphate stabilizer (E-450i), along with salt, water, and oil, was purchased from a local supermarket in Valencia, Spain. To obtain BBP, beetroot was processed in an electric food processor (DeLonghi, Barcelona, Spain) for 10 s to extract the juice, replicating industrial-scale procedures [53].

### 3.2. Preparation of Beetroot By-Product

To produce BBP, the raw material was first distributed on aluminum trays and frozen for 24 h at −45 °C in a vertical freezer (CVF450/45, Ing. Climas, Barcelona, Spain). Then, samples were freeze-dried using a Lioalfa-6 Lyophilizer (Telstar, Spain) at 2600 Pa and −56.6 °C for 48 h. The dried product was subsequently ground with a grinder (Minimoka, Taurus, Lleida, Spain) for 1 min at 25 °C and sieved through a 200 μm mesh to yield a free-flowing powder with a moisture content of 2%.

### 3.3. Gluten-Free Bread Dough Formulation

Four types of gluten-free bread dough were prepared, differentiated by leavening system—baking powder (BP) and baker’s yeast (BY)—and BBP addition (4%). Base ingredients remained constant across all formulations: 56% water, 0.4% salt, and 1.2% oil. The gluten-free flour blend was adjusted to compensate for BBP addition, ranging from 36% to 41.52% depending on the formulation. Leavening agents were used at 2.4% for BP formulations and 0.88% for BY formulations. Ingredients were mixed with a kneader (Kenwood Chef Classic, KM400/99 plus, Kenwood Corporation, Tokyo, Japan). The kneading process started with 45 s at minimum speed, then continued for 5 min at the speed set at 2. After mixing, the dough with baker’s yeast was allowed to ferment for 30 min in an incubator, then was cooled to 25 °C (FOC 225I, VELP Scientifica, Italy). The prepared dough was then loaded into a syringe for the 3D printing process. Each formulation was prepared in three independent batches.

### 3.4. 3D-Printing Process

A Moore 1 Mini Clay 3D printer (Shenzhen Tronxy Technology Co., Ltd., Shenzhen, China) was employed to print gluten-free dough, both with and without beetroot by-product. The printing utilized Fused Deposition Modeling (FDM) technology, featuring a precise X-Y-Z positioning system and stepper motor-controlled extrusion. Shapes, including a rectangle and an oval (70 × 30 × 20 mm), were designed in Tinkercad (Autodesk, San Rafael, CA, USA), and Ultimaker Cura (version 5.1.1, by Ultimaker B.V, Brooklyn, NY, USA) was used to set the printing parameters. The settings included a 60% rectilinear infill, a layer height of 1.7 mm, and a print speed of 25 mm/s. All samples were printed with a 1.2 mm diameter nozzle at 25 °C. The printed samples were baked at 190 °C for 17 min in a Convotherm Mini convection oven (Welbilt Iberia, Barcelona, Spain). Following baking, samples were cooled to 25 °C before analytical determinations.

### 3.5. Analysis

All analyses were performed in triplicate.

#### 3.5.1. Moisture Content and pH Determination

Moisture and pH were evaluated in the bread dough, crust, and crumb of 3D-printed gluten-free bread. The moisture content of the samples was determined by vacuum drying in an oven (Vaciotem, J.P. Selecta, Spain) at 70 ± 1 °C and a pressure below 100 mmHg until a constant weight was reached [54]. pH values were measured in triplicate using a HI99163 pH meter (Hanna Instruments Inc., Woonsocket, RI, USA).

#### 3.5.2. Color Analysis

The color of the dough, crust, and crumb of 3D-printed gluten-free bread was determined by the CIEL*a*b* color space method. Color coordinates were obtained using a Konica Minolta CM-700d colorimeter (Konica Minolta CM-700d/600d series, Tokyo, Japan) with standard illuminant D65 and a visual angle of 10°. Results were obtained in terms of L* (brightness: L* = 0 (black), L* = 100 (white)), a* (−a* = green, +a* = red), and b* (−b* = blue, +b* = yellow), according to the CIEL*a*b* system [55]. Also, the difference in color between the samples was evaluated. Color differences were evaluated between leavening agents (baking powder vs. baker’s yeast) and between geometries (rectangular vs. oval) for each formulation.

#### 3.5.3. Bioactive Compounds

Spectrophotometric determination of betalains (betacyanins and betaxanthins) was performed according to Nilsson [56], following the same procedure as Igual et al. [5]. Samples were mixed with a phosphate buffer (0.05 M, pH 6.5). The absorbance of samples was measured at 476 (betacyanin), 535 (betaxanthin), and 600 (correction) nm with a phosphate buffer used as a blank. Results were expressed as mg betanin equivalents per 100 g dry matter (mgBE/100 g_dry solid_) for betacyanins and mg vulgaxanthin-I equivalents per 100 g dry matter (mgVE/100 g_dry solid_) for betaxanthins.

Total phenols were assessed using the Folin–Ciocalteu colorimetric assay as outlined by Igual et al. [5]. Methanol was used for the sample extraction. Phenolic content was quantified against a gallic acid calibration curve and reported as mg gallic acid equivalents per 100 g dry matter (mg GAE/100 g_dry solid_).

Antioxidant capacity was evaluated using the DPPH method, as described by Igual et al. [5]. Methanol was used for the sample extraction. Results were expressed as mg Trolox equivalents per 100 g dry matter (mg TE/100 g_dry solid_).

### 3.6. Statistical Analysis

A multifactorial ANOVA was applied to evaluate the effect of the studied factors and their interactions. Model refinement was carried out by stepwise exclusion of non-significant terms (*p* < 0.05), resulting in a reduced model containing only statistically significant main effects and interactions. The content of betalains (betanin and vulgaxanthin) was determined exclusively in formulations enriched with BBP, as these pigments are not present in the control samples. Therefore, a simple analysis of variance (one-way ANOVA) was performed to evaluate the effect of the leavening agent on the retention of these bioactive compounds. Fisher’s post hoc least significant differences (LSDs) were applied to establish significant statistical differences between samples. All statistical computations were performed using XLSTAT 2024.3.0 software (Lumivero, 2023), applying two-tailed tests with a significance threshold of α = 0.05.

## 4. Conclusions

The incorporation of BBP into 3D-printed gluten-free bread improved its nutritional profile by increasing the content of bioactive compounds (TP, AC, and betalains), thereby adding value to a by-product of the beetroot food industry.

Depending on the type of agent used, the bread exhibited different behaviors. Breads made with baker’s yeast and BBP had a higher bioactive profile in both the crust and crumb, with higher TPC, AC, and betalain content. Betanins were the predominant pigments in the crumb, responsible for the red-violet coloration. In contrast, breads made with baking powder showed lower levels of bioactive compounds, although they had higher concentrations of vulgaxanthin in the crumb. This behavior is attributed to the more alkaline pH of baking powder, which favors the stability of vulgaxanthin and gives the product a more yellowish color. Geometry had a minor but significant influence on the final properties. Oval samples with BBP had higher moisture content in both the crust and crumb, due to their smaller surface area exposed to heat during baking. This geometry also favored better TP and AC retention. Rectangular geometry showed only higher vulgaxanthin values. Correlation analyses revealed that pH is a critical factor determining the stability of betalains, as lower pH values favor the preservation of betanin. In addition, the distribution of bioactive compounds differed significantly between the crust and the crumb. The crust, being more exposed to heat, generally had lower concentrations, which favors the degradation of heat-sensitive compounds during baking. Based on this study, formulations with baker’s yeast, oval geometry, and the addition of BBP can be recommended.

This study demonstrates the development of gluten-free bakery products enriched with beet by-products using 3D printing technology, which enables the optimization of nutritional profiles by controlling variables such as the type of leavening agent and geometric design.

## Figures and Tables

**Table 1:** Mean values (and standard error) of pH, moisture (%) and color parameters (L*, a* and b*) of gluten-free dough as affected by leavening agent and beetroot by-product addition.

	pH	Moisture (%)	L*	a*	b*
**Leavening Agent**					
Baking Powder	7.037 (0.012)	60.2 (0.2) ^b^	59 (11)	15 (8)	6.5 (0.8) ^b^
Baker’s Yeast	5.81 (0.02)	61.48 (0.04) ^a^	60 (10)	16 (8)	7.3 (0.7) ^a^
**Beetroot by-product**					
With BBP	6.4 (0.2)	60.7 (0.3)	36.8 (0.6)	32.9 (0.5)	5.2 (0.3) ^b^
Without BBP	6.4 (0.2)	61.2 (0.2)	82.5 (0.4)	−1.7 (0.2)	8.6 (0.2) ^a^
**Leavening Agent × BBP**					
BP × N-BBP	7.063 (0.007) ^a^	60.49 (0.14)	83.3 (0.2) ^a^	−2.25 (0.02) ^d^	8.3 (0.2)
BP × Y-BBP	7.022 (0.005) ^b^	60.2 (0.2)	35.47 (0.02) ^d^	31.7 (0.2) ^b^	4.60 (0.03)
BY × N-BBP	5.762 (0.005) ^d^	61.50 (0.02)	81.7 (0.2) ^b^	−1.22 (0.05) ^c^	8.87 (0.13)
BY × Y-BBP	5.843 (0.008) ^c^	61.46 (0.08)	38.02 (0.06) ^c^	34.14 (0.14) ^a^	5.8 (0.2)

Different letters (a–d) within the same column indicate the significant differences (*p* < 0.05) according to Fisher’s LSD post hoc test. Letters are shown only for factors or interactions that were statistically significant in the multifactorial ANOVA. BP: Baking powder; BY: baker’s yeast; BBP: beet by-product; N-BBP: without beet by-product; Y-BBP: with beet by-product.

**Table 2:** Images of crumb from 3D-printed gluten-free bread as affected by leavening agent (baking powder vs. baker’s yeast), geometry (R: rectangular; O: oval), and beetroot by-product (BBP) addition.

	Crumb			
	Baking Powder		Baker’s Yeast	
	R	O	R	O
Without BBP	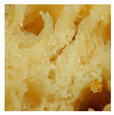	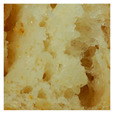	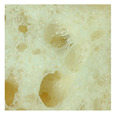	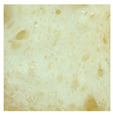
With BBP	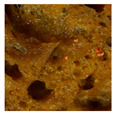	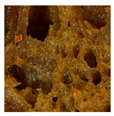	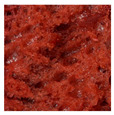	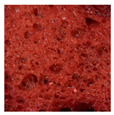

**Table 3:** Mean value (and standard error) total phenol content (TPC, mgGAE/100g dry solid) and antioxidant capacity (AC, mgTE/100g dry solid) of gluten-free bread as affected by leavening agent, shape and beetroot by-product addition (BBP).

	Crust		Crumb	
	TPC	AC	TPC	AC
**Leavening Agent × Shape**				
BP × R	21.1 (0.4) ^c^	5 (3)	63.3 (1.3)	7 (4) ^c^
BP × O	23.1 (1.3) ^b^	5 (3)	78 (5)	8 (5) ^c^
BY × R	26 (2) ^a^	9 (5)	72.1 (1.6)	14 (6) ^b^
BY × O	23.6 (0.9) ^b^	11 (4)	96 (10)	19 (7) ^a^
**Leavening Agent × BBP**				
BP × N-BBP	23.5 (1.2) ^b^	0 (0)	64.5 (1.4)	0 (0) ^d^
BP × Y-BBP	20.7 (0.3) ^c^	9.9 (0.6)	77 (5)	14.9 (0.8) ^b^
BY × N-BBP	22.5 (0.5) ^b,c^	2.2 (1.2)	71.7 (1.5)	4.8 (1.3) ^c^
BY × Y-BBP	27 (2) ^a^	18.1 (0.4)	96 (9)	27.4 (1.8) ^a^
**Shape × BBP**				
R × N-BBP	21.4 (0.4) ^b^	0.11 (0.07)	65 (2)	1.3 (0.8)
R × Y-BBP	26 (2) ^a^	14 (2)	70 (2)	19 (3)
O × N-BBP	24.6 (0.7) ^a^	2.1 (1.2)	71 (2)	3 (2)
O × Y-BBP	22.1 (1.2) ^b^	14 (2)	103 (6)	23 (4)
**Leavening Agent × Shape × BBP**				
BP × R × N-BBP	21.2 (0.9)	0 (0) ^d^	61.5 (0.3) ^f^	0 (0)
BP × O × N-BBP	25.8 (0.8)	0 (0) ^d^	67.5 (0.2) ^d,e^	0 (0)
BP × R × Y-BBP	21.2 (0.5)	10.06 (1.06) ^b^	65 (2) ^e^	13.80 (0.12)
BP × O × Y-BBP	20.38 (0.02)	9.9 (0.9) ^b^	89.1 (1.2) ^b^	16.2 (0.9)
BY × R × N-BBP	21.6 (0.2)	0.21 (0.08) ^d^	69.1 (1.9) ^d^	2.6 (0.7)
BY × O × N-BBP	23.4 (0.4)	4.3 (0.3) ^c^	74.3 (0.8) ^c^	6.9 (0.9)
BY × R × Y-BBP	30.7 (0.4)	18.1 (0.2) ^a^	75.2 (0.3) ^c^	24.4 (0.3)
BY × O × Y-BBP	23 (2)	18.1 (0.9) ^a^	116.9 (0.5) ^a^	30.3 (0.9)

Different letters (a–f) within the same column indicate the significant differences (*p* < 0.05) according to Fisher’s LSD post hoc test. Letters show only interactions that were statistically significant in the multifactorial ANOVA. BP: Baking powder; BY: baker’s yeast; BBP: beet by-product; R: rectangular; O: oval; N-BBP: without beet by-product; Y-BBP: with beet by-product.

**Table 4:** Betalain (betanin and vulgaxanthin) retention (%) in crust and crumb of 3D-printed gluten-free bread with beetroot by-product relative to raw dough, as affected by leavening agent and geometry.

	Betanin Retention (%)		Vulgaxanthin Retention (%)	
Sample	Crust	Crumb	Crust	Crumb
BPR	20.3	38.3	26.7	57.4
BPO	21.8	32.6	27.2	49.6
BYR	21.6	39.8	21.0	35.9
BYO	22.2	38.8	19.1	33.3

BPR: Baking powder and rectangular geometry; BPO: baking powder and oval geometry; BYR: baker’s yeast and rectangular geometry; BYO: baker’s yeast and oval geometry.

## Data Availability

The original contributions presented in this study are included in the article. Further inquiries can be directed to the corresponding author(s).

## Abbreviations

The following abbreviations are used in this manuscript:

BBP	Beetroot by-product
BP	Baking powder
BY	Baker’s yeast
TPC	Total phenol content
AC	Antioxidant capacity
R	Rectangular
O	Oval
N-BBP	Without beet by-product
Y-BBP	With beet by-product
